# Dorsal Root Ganglion Stimulation for Postherpetic Neuralgia: A Pilot Scoping Review of the Current Evidence

**DOI:** 10.7759/cureus.93664

**Published:** 2025-10-01

**Authors:** Adam Dorner, Iulia Dascalu, Jill Osborn, Vishal Varshney

**Affiliations:** 1 Faculty of Medicine, University of British Columbia, Vancouver, CAN; 2 Department of Anesthesiology, Pharmacology, and Therapeutics, University of British Columbia, Vancouver, CAN; 3 Department of Anesthesia, Providence Health Care, University of British Columbia, Vancouver, CAN

**Keywords:** dorsal root ganglion stimulation, neuromodulation, neuropathic pain, pain management, postherpetic neuralgia

## Abstract

This review synthesizes current evidence regarding the use of dorsal root ganglion (DRG) stimulation, a form of pain neuromodulation, for managing postherpetic neuralgia (PHN), a debilitating chronic neuropathic pain syndrome arising from herpes zoster virus infection. Comprehensive searches across multiple databases (MEDLINE, Embase, Cumulative Index to Nursing and Allied Health Literature (CINAHL), Cochrane Library) for studies published from 2010 onward were supplemented by manual reference checks and gray literature review. Inclusion criteria encompassed adults with PHN (pain persisting ≥ three months post-rash onset) treated with DRG stimulation involving varying lead placements and stimulation techniques. Two independent reviewers screened articles and extracted data, resolving disagreements through consensus or a third reviewer, with a Preferred Reporting Items for Systematic Reviews and Meta-Analyses (PRISMA) flow diagram documenting study selection, resulting in 23 included studies after screening 117 initial references. Extracted data revealed variability in DRG stimulation techniques (percutaneous vs. surgical lead placement) and parameters (tonic, burst, high-frequency), with reported outcomes including pain scores, quality of life, opioid use, and complications. While case series support DRG efficacy for PHN, studies are limited by small sample sizes and a lack of prospective or randomized trials focused exclusively on PHN. Many studies included mixed-pain populations, complicating direct comparisons. Broader neuromodulation evidence suggests DRG may cause fewer paresthesias than spinal cord stimulation (SCS). Some studies indicate DRG may be more effective for other neuropathic pain types than for PHN. This review identifies significant variability and substantial gaps in the literature, notably the absence of rigorous randomized controlled trials and studies focusing solely on PHN, underscoring the need for standardized protocols and targeted clinical trials. Although DRG stimulation shows potential for managing PHN, its comparative effectiveness relative to other pain etiologies warrants further investigation to refine treatment approaches and optimize patient outcomes.

## Introduction and background

Chronic pain is a widespread, debilitating health issue that severely impairs quality of life. One significant form of chronic neuropathic pain is postherpetic neuralgia (PHN), which develops following reactivation of the varicella-zoster virus (herpes zoster or “shingles”). PHN often persists well after the resolution of the initial rash and can lead to lingering discomfort that negatively affects daily functioning and overall well-being [1].

Neuromodulation techniques, such as spinal cord stimulation (SCS), have been employed for decades to address various neuropathic pain conditions, including PHN [2]. However, dorsal root ganglion (DRG) stimulation has more recently emerged as a potentially superior and more targeted approach to manage specific pain states; by precisely modulating sensory nerve signals within the ganglia, DRG stimulation may offer enhanced pain relief with fewer unwanted paresthesias compared to conventional SCS, especially in focal or dermatomally concentrated pain syndromes like PHN [3]. Despite promising early reports, the evidence base for DRG stimulation in PHN is still evolving, with significant variability in patient populations, procedural protocols, and outcome measures across studies.

Given the limited and heterogeneous nature of available studies, a pilot scoping review is warranted to provide a foundational mapping of the current literature on DRG stimulation for PHN. This review seeks to chart the breadth and quality of existing evidence, highlight key knowledge gaps, and clarify the characteristics of interventions and reported outcomes. By serving as an initial synthesis in this evolving field, our pilot review establishes a baseline for future, more definitive research and aims to guide the direction of upcoming clinical investigations and evidence-based practice.

## Review

Methods

This scoping review was conducted following the Joanna Briggs Institute (JBI) methodology. A review protocol for this scoping review was published to the Open Science Framework on February 15, 2025 (Registration address: https://doi.org/10.17605/OSF.IO/DKNFP). Comprehensive searches were performed across multiple databases (MEDLINE, Embase, Cumulative Index to Nursing and Allied Health Literature (CINAHL), Cochrane Library) for literature published from 2010 onward. The most recent search was executed on January 22, 2025. Initial searches yielded 117 references, supplemented by manual reference checks and a gray literature review to ensure comprehensive coverage. Table 1 contains the full electronic search strategy for MEDLINE, with equivalent strategies adapted for the other databases.

**Table 1:** Ovid MEDLINE search strategy.

MEDLINE (Ovid) search strategy	
Line	Ovid MEDLINE search string
1	exp Neuralgia, Postherpetic/ or (postherpetic neuralgia or post-herpetic neuralgia or phn).mp.
2	exp Ganglia, Spinal/ or (dorsal root ganglion or dorsal root ganglia or drg).mp.
3	exp Electric Stimulation Therapy/ or exp Spinal Cord Stimulation/ or (drg stimulation or dorsal root ganglion stimulation or neurostimulation or neuromodulation).mp.
4	2 and 3
5	1 and 4

**Figure 1:**
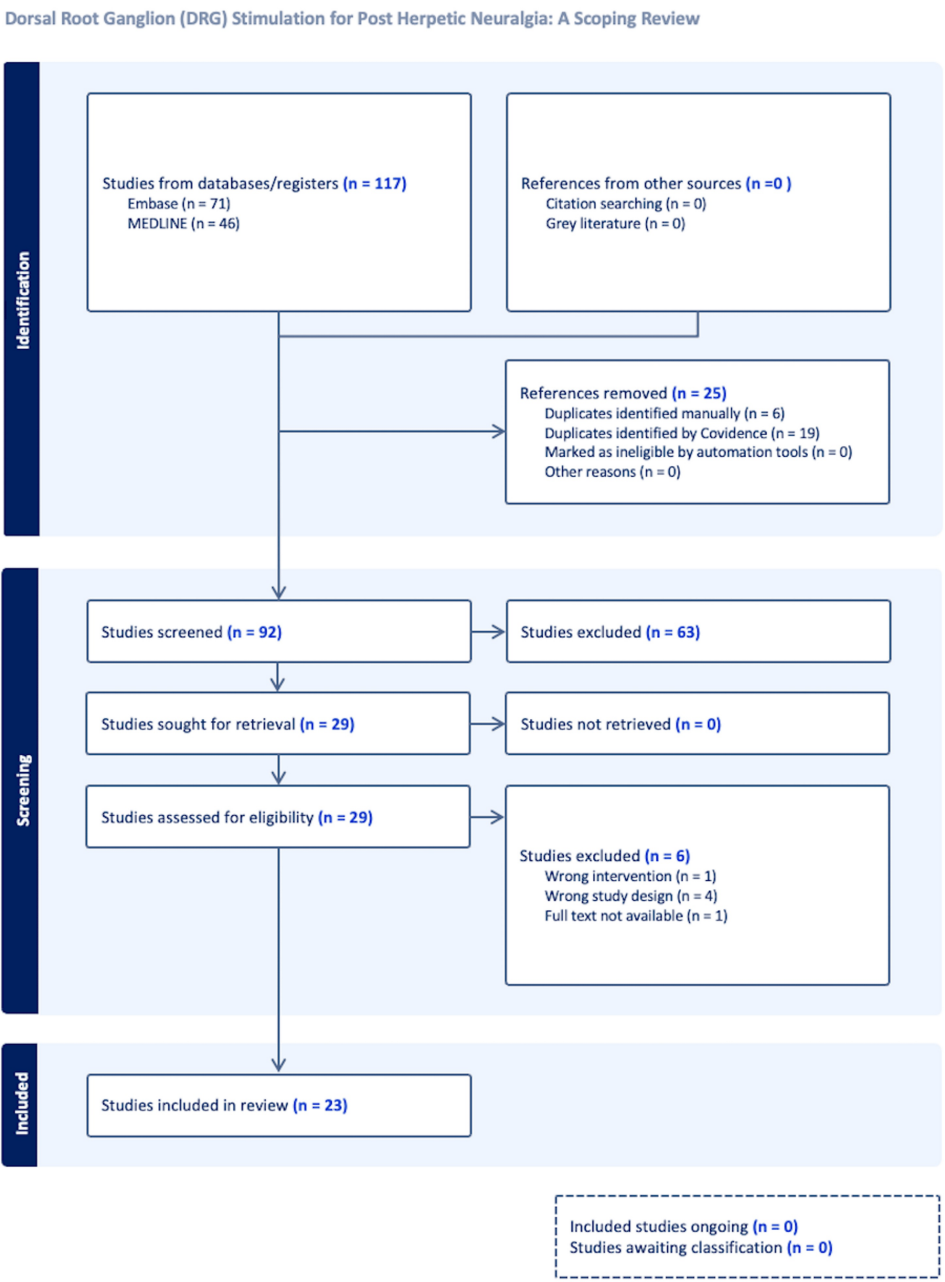
Preferred Reporting Items for Systematic Reviews and Meta-Analyses (PRISMA) flowchart.

Two independent reviewers screened articles based on titles and abstracts, applying predefined inclusion criteria: adults diagnosed with PHN (≥ three months duration), receiving DRG stimulation treatment. A third reviewer addressed disagreements and made final decisions on study inclusions and exclusions. Full-text reviews were conducted for eligible studies, with disagreements resolved by consensus or consultation with a third reviewer. Studies that met the inclusion criteria and appeared in the literature search but were published as abstracts without available full text were also included. This rigorous screening process resulted in the inclusion of 23 relevant studies, detailed in a Preferred Reporting Items for Systematic Reviews and Meta-Analyses (PRISMA) flow diagram (Figure 1).

For each included study, the following data were systematically extracted using a standardized template: study citation (author, year, journal), country and clinical setting, study design, population and participant characteristics (including sample size, demographics, and inclusion criteria), duration of pain, details of the DRG intervention (e.g., percutaneous or surgical approach, targeted DRG levels, and lead configuration), stimulation parameters/programming (e.g., type, frequency, amplitude, and pulse width), comparator (if applicable), outcomes measured (pain intensity, functional scores, opioid consumption, quality of life, adverse events/complications), key findings/results (including effect sizes and statistical significance where reported), adverse events/complications, length of follow-up and maintenance/durability, author conclusions, study limitations or bias, relevance to the review question, and any other relevant notes. All data variables were extracted as reported in the source publications; where data were missing or unclear, this was noted in the extraction form.

No formal critical appraisal of individual studies was performed, in keeping with the scoping review methodology. The aim was to comprehensively map the existing literature on DRG stimulation for PHN regardless of study quality, to capture the breadth of available evidence and identify knowledge gaps. This approach allows for the inclusion of diverse study designs and ensures a broad overview of current research, which is particularly valuable in areas with limited and heterogeneous literature.

Results

In total, 23 studies investigating DRG stimulation for the management of PHN were included. The reviewed studies varied significantly in design, encompassing case reports, retrospective case series, observational studies, and limited randomized controlled trials (RCTs). Below, key findings, outcomes, and limitations of all studies are detailed, organized by study design.

Case Reports

Several case reports demonstrated promising results for DRG stimulation in managing refractory PHN. Bara et al. (2015) reported on a single patient (51-year-old female) whose pain intensity, measured by the Visual Analog Scale (VAS), reduced notably from 90 mm at baseline to 30 mm within two weeks post implant. No complications were reported; however, the study's single-case nature and short-term follow-up limited generalizability [4]. Similarly, Lynch et al. (2011) documented substantial pain relief in an 80-year-old patient whose Numerical Rating Scale (NRS) scores decreased from constant severe pain at baseline to mild, intermittent pain post treatment. Specifically, after seven days of trial stimulation, pain had decreased to 50% of the pre-implantation level, activities of daily living had increased, and the patient had stopped all pain medications. Following permanent implantation, at six-month follow-up, the patient's pain had decreased to 20% of its pre-implantation level, and the patient had stayed off pain medications. No adverse reactions were reported. Again, the single case nature of this study limits generalizability [5].

Johnson et al. (2021) described the successful surgical implantation of DRG leads in a 65-year-old patient with PHN who had failed previous therapies, including an intrathecal pump, percutaneous and paddle dorsal column stimulators, and individual nerve root blocks. The study reported a technical success of an open surgical placement of left-sided T9-T12 DRG stimulators, with laminectomies and foraminotomies performed to expose the intervertebral foramina and DRG electrodes being inserted into the foramina at T9-T12. Although the patient experienced a transient improvement in neuropathic pain, there was no long-term pain relief [6].

Hong et al. (2021) utilized combined DRG and SCS in a 54-year-old male. This resulted in a >50% reduction of PHN pain during the one-week trial as well as ongoing significant pain relief at a two-month follow-up, highlighting potential synergistic effects [7]. Similarly, in a 2023 case report by Wang et al., a 71-year-old male with PHN was treated using a novel dual-function DRG stimulator capable of delivering both DRG and pulsed radiofrequency neuromodulation. The system allowed flexible targeting of the patient's thoracic dermatomal pain distribution. The patient experienced a substantial reduction in pain intensity, from 8/10 to 2/10 on the NRS prior to discharge, with no reported complications. There was enduring relief and functional improvement at a three-month follow-up appointment [8]. These cases highlight the potential benefit of dual-function systems in managing complex or treatment-resistant PHN.

Case Series, Retrospective, and Cross-Sectional Studies

Numerous retrospective and case series studies provided broader insights. Sullivan et al. (2015), in a retrospective Australian series, reported significant mean pain reductions from baseline VAS scores of approximately 77 mm down to 37 mm during the trial period among 11 patients with mixed pain etiologies, despite noting three failed trials. Three patients had >80% pain relief at the end of the trial. At one and three months post implant, patients reported 59.3% ± 37.6% (N = 5) and 58.9% ± 36.9% (N = 5) improvement in their pain, respectively. However, the study population only included one patient with PHN, and pain scores for individual patients were not reported [9].

Vesper et al. (2016) described 11 patients undergoing cervical and upper thoracic DRG stimulation for mixed chronic pain etiologies, with nine reporting clinical improvements. Mean VAS scores decreased from 8.1 to 2.3 over the course of the trial period, which ranged from three to seven days with external electrodes. However, precise quantitative outcome data were sparse, and the limited specificity regarding the exact number of PHN cases impaired the direct applicability to PHN patients. Of note, the one patient who did not have clinically significant pain reduction after implantation was a patient with PHN [10].

Vesper et al. (2017) evaluated outcomes in a larger cohort of chronic pain patients (n = 114) through a retrospective chart review, reporting a high rate (83%) of trial-to-permanent implantation. While the mean pain intensity (VAS) was significantly reduced amongst the group, on average from 8.1 to 3 at 24 months, 18 patients required device explantation due to loss of efficacy or complications such as infection. However, there was no specific subgroup analysis for PHN patients, nor was the exact number of PHN patients included specified, limiting its applicability to this study [11].

Anthony et al. (2019) examined six patients with thoracic neuralgia (TN) of various etiologies, including post-mastectomy, post-abdominoplasty, and PHN; these patients underwent trial DRG stimulation. The average baseline pain score was 6.8 (VAS, 0-10). Only two of the six patients (33%) experienced meaningful pain relief and proceeded to permanent implantation, with both reporting VAS scores of 0 and 1, and complete discontinuation of opioid use; neither of these was a PHN patient. In fact, all three patients with PHN (ages 46, 68, and 76, all male) failed to respond to DRG stimulation during the trial phase; two reported no pain improvement, and while one had some pain improvement with a 31% VAS reduction, this patient required device removal due to pain caused by leads during the trial period [12]. While DRG stimulation may be beneficial for TN of some etiologies, these findings suggest it may be less effective for PHN.

Pajuelo et al. (2019) provided multicenter retrospective data involving 22 PHN patients. At baseline, all patients reported high pain intensity. Patients were implanted with a quadripolar lead placed over the dorsal aspect of DRG using a percutaneous approach, and at a six-month follow-up, 60% of patients reported lower pain intensity scores, with improved quality of life scores and reduced allodynia reported as well [13]. This study was limited by its retrospective design and small sample size.

Papa et al. (2020) reviewed outcomes for 44 patients with chronic neuropathic pain of several etiologies, noting substantial pain relief, but adverse events, including lead migration and infection, occurred in approximately 20% of patients, indicating notable procedural risks. Out of the 44 patients, 39 patients (89%) were responsive to a DRG stimulation trial and had an internal pulse generator implanted; the remaining five non-responsive patients were explanted with the trial device. For the 39 implanted patients, the average degree of pain relief was 72% on the VAS scale during the trial, compared to 11% in patients who did not undergo the final system implantation. For those implanted, average pain relief at 48 months was 74.1%. Five PHN patients were included in the study population, and the authors noted that the PHN subgroup had a lower responder rate to DRG stimulation for pain relief compared to other chronic pain etiologies [14].

Placeway et al. (2020) and Kim et al. (2020, 2021) reported on small cohorts of patients with PHN (two to three patients), achieving marked pain improvements (VAS scores reduced by 50-98%) both during a short-term trial phase and over the course of long-term follow-up (12-36 months) with minimal reported adverse events. Placeway et al. (2020) reported one patient also significantly reducing oral painkiller use after DRG implantation, noting another potential benefit of this method of analgesia [15-17]. Nonetheless, these studies highlighted small sample limitations affecting reliability.

In a 2024 case series by Verma et al., three patients with refractory PHN underwent DRG stimulation (DRGS) and experienced greater than 40% pain reduction per their VAS scores at 12 months post implant. Improvements were also observed in functional status and mental health, as measured by the Pain Disability Index (PDI), the nine-item Patient Health Questionnaire (PHQ-9), and the Generalized Anxiety Disorder scale (GAD-7), along with reduced reliance on analgesic medications [18].

In a retrospective single-center study of seven patients with refractory PHN by Isagulyan et al. (2024), DRGS using standard SCS systems resulted in a mean VAS reduction of 62.3% at 12 months. Four of the patients experienced good to excellent pain relief, defined as a VAS reduction of >50%, and improved quality of life. However, electrode displacement occurred in two patients requiring the implantation of an alternate system or necessitating surgical correction of electrode position [19]. The small sample size and use of non-dedicated DRGS systems limit generalizability.

In a 2022 case series by Semenov et al., six patients (median age of 74 years) with refractory PHN underwent DRG stimulation using standard SCS systems due to the unavailability of DRG-specific devices. Electrodes were placed under X-ray guidance, with follow-up conducted over a median of two years. Median baseline pain intensity was 9.08 (modified Brief Pain Inventory), which decreased to 3.42 post treatment, a 62.3% reduction. Three patients experienced “great” recovery (≥75% pain reduction), one had “good” recovery (50-75%), one had “moderate” recovery (25-50%), and one had “unsatisfactory” recovery (<25%). Two cases of lead dislocation occurred, attributed to a lack of proper fixation systems, but no major complications were reported. Most patients reduced or discontinued pain medication, and nearly all reported improved daily functioning and sleep quality [20].

Maiti et al. (2023) examined 21 patients with various chronic pain conditions, including PHN, noting substantial improvements in quality-of-life metrics and pain intensity, although there was sparse quantitative long-term follow-up data, limiting long-term conclusions [21]. A second study by Maiti et al. (2023) reported on three patients with refractory PHN who experienced significant pain relief and improved quality of life following DRG stimulation. Alongside reductions in medication use and VAS pain scores, improvements were also observed in anxiety, depression, and pain-related disability scales [22]. While the findings support DRG as a promising targeted therapy for PHN, the study is limited by its small sample size and case report design, as well as a lack of reported quantitative data.

A 2021 study by Parker et al. examined cortical activity in 13 chronic pain patients (including one with PHN) during DRGS ON and OFF conditions, using power spectral analysis to correlate brain oscillations with pain levels. Pain relief was associated with a shift from low-frequency theta to higher-frequency low beta activity, suggesting a potential biomarker of DRGS efficacy [23]. Although not a direct measure of pain, this approach offers insight into the central mechanisms underlying DRGS. In the context of PHN, only one patient was included, and individual results were not reported separately. While promising, these findings are not directly applicable to PHN due to the limited representation and lack of subgroup data.

Prospective Studies and Trials

Limited prospective evidence was available. Eldabe et al. (2022), in a prospective cohort study of 42 patients, where 32 received DRG implants following a trial, included two with PHN. However, PHN-specific outcomes were not reported separately. Among those who retained the device, pain intensity decreased with a mean difference of 1.7 points on the VAS at 24 months. Quality-of-life (EuroQol 5-Dimensions 3 Level, EQ-5D-3L) scores also improved modestly, with a statistically significant change observed at the last follow-up. Lead migration, infection, and other factors necessitated procedural revisions in 14 patients, highlighting ongoing safety concerns [24]. Notably, study attrition led to only 22 patients reporting a full set of data. Two PHN patients were included in this study, but data for this subgroup were not reported separately, limiting applicability.

Piedade et al. (2023) conducted a randomized double-blind crossover trial involving 17 patients with implanted DRG stimulation systems and evaluated the effects of different stimulation frequencies (4 Hz, 20 Hz, 60 Hz, and sham) on pain and quality-of-life outcomes. These patients had various pain etiologies. Each frequency was applied for five days with two-day washout periods in between. Pain intensity was lowest with 4 Hz stimulation (VAS 3.8), compared to 20 Hz (VAS 4.2), 60 Hz (VAS 4.6), and sham (VAS 5.3), though only the comparison between 4 Hz and sham reached statistical significance. Trends favoring 4 Hz were also seen on depression scores, while quality-of-life and pain questionnaire outcomes slightly favored 20 Hz, which the paper mentions as the current standard frequency for DRGS. The authors concluded that low-frequency stimulation did not significantly outperform standard settings, possibly due to limited sample size and short observation periods. Of note, only one patient in the cohort had PHN; the PHN patient’s VAS remained relatively low throughout, scoring at 2-3, though the 20 Hz condition gave a higher VAS of 5 [25].

Parker et al. (2021) contributed a unique phase I/II randomized, single-blinded, sham-controlled trial in 16 patients, including two PHN patients, examining possible synergistic effects of DRGS and transcranial direct current stimulation (tDCS). DRGS-ON alone resulted in a 37% mean reduction in pain, while DRGS-ON paired with active tDCS resulted in a 57% mean reduction in pain compared to DRGS-OFF, again highlighting synergistic effects. However, again, the study’s small sample and heterogeneous pain etiology group limit generalizability to PHN patients (Table 2) [26].

**Table 2:** Summary of results. PHN: postherpetic neuralgia; VAS: Visual Analog Scale; DRG: dorsal root ganglion; SCS: spinal cord stimulation; NRS: Numerical Rating Scale; QoL: quality of life; DRGS: dorsal root ganglion stimulation; tDCS: transcranial direct current stimulation; IPG: implantable pulse generator.

Citation	Design	Sample size (PHN)	Outcome	Side effects
Anthony et al. (2019) [12]	Retrospective chart review	N=6 (PHN=3)	• No meaningful pain relief for PHN patients	• 1 device removed (lead pain). Others: minimal/no benefit
Bara et al. (2015) [4]	Case report	N=1 (PHN=1)	• VAS 90→30 (67% ↓), improved function	• None
Eldabe et al. (2022) [24]	Prospective study (multi-etiology)	N=42 total (PHN=2)	• 22/42 completed → mean VAS ↓ 1.7 at 2 years. PHN data not reported separately	• 5 explants (lead pain or lack of benefit). Other: infection, lead migration/fracture, IPG issues
Hong et al. (2021) [7]	Case report	N=1 (PHN=1)	• Combined DRG + SCS. >50% pain ↓ during trial	• None
Isagulyan et al. (2024) [19]	Retrospective case series	N=7 (PHN=7)	• Mean VAS ↓ 62% at 1 year (3.4 average)	• 2 unsatisfactory outcomes (incl. postop complications)
Vesper et al. (2016) [10]	Case series (multi-etiology)	N=11 total (PHN=1)	• 9/11 implanted, but PHN patient: no sustained relief	• 1 transient arm/hand paresis. Frequent reprogramming needed
Johnson et al. (2021) [6]	Case report (technical focus)	N=1 (PHN=1)	• Surgical technique study, stable electrode placement achieved, but only transient pain relief	• Extensive epidural scarring
Kim et al. (2021) [17]	Case series	N=3 (PHN=3)	• All had sustained pain ↓ (>50%), NRS: 8→2–5. Improved function/QoL	• Pt 1: infection/erosion → explant + reimplant. Pt 2: lead dislodgement → revision
Lynch et al. (2011) [5]	Case report	N=1 (PHN=1)	• Major pain ↓ after trial. Discontinued all meds	• None reported
Maiti et al. (2023) (case series, multi-etiology) [21]	Case series (multi-etiology)	N=21 (PHN not specified)	• 85% overall had sig. pain improvement. PHN subgroup outcomes are not reported separately	• Lead migration (1). 2 explants (MRI requirement)
Maiti et al. (2023) (PHN-only) [22]	Case series	N=3 (PHN=3)	• All had significant pain relief, improved QoL reported	• None mentioned
Pajuelo et al. (2019) [13]	Multicenter retrospective study	N=22 (PHN=22)	• 60% had significant pain ↓ by 6 months	• Not reported
Papa et al. (2020) [14]	Retrospective data collection (multi-etiology)	N=44 (PHN=5)	• Overall: 74% pain ↓ at 48 months in those implanted, but PHN patients had “less response”	• Lead migration/infection in ~20%
Parker et al. (2021) (MEG study) [23]	Cross-sectional study (multi-etiology)	N=13 total (PHN=1)	• Acute pain ↓ 21% with DRGS ON; PHN data not separated	• Not reported
Parker et al. (2021) (tDCS + DRGS trial) [26]	Sham-controlled crossover trial (multi-etiology)	N=16 total (PHN=2)	• DRGS alone ↓ pain 37%; DRGS + active tDCS ↓ pain 57%; PHN subgroup showed similar benefit	• Mild tDCS-related (itching, burning, tingling); all tolerable
Piedade et al. (2023) [25]	Randomized double-blind crossover trial (multi-etiology)	N=17 total (PHN=1)	• PHN pt: baseline VAS 2. At 4 Hz VAS 3. At 20 Hz VAS 5. At 60 Hz VAS 2. Sham VAS 3	• Not reported
Placeway et al. (2020) [15]	Case reports	N=2 (PHN=2)	• Case 1: ~98% pain relief at 3 years. Case 2: 80% pain relief at 2 years	• None
Semenov et al. (2022) [20]	Case series	N=6 (PHN=6)	• Median pain ↓ 62% post treatment	• 2 lead dislocations (1 converted to burst-SCS)
Sullivan et al. (2015) [9]	Retrospective case series (multi-etiology)	N=11 total (PHN=1)	• At 1–3 months: ~59% ↓ pain, but PHN outcomes not separated	• Not specified
Verma et al. (2024) [18]	Retrospective case series	N=3 (PHN=3)	• All had >40% pain ↓ at 12 months	• None
Vesper et al. (2017) [11]	Retrospective chart review (multi-etiology)	N=114 total (PHN included, number not given)	• Mean VAS ↓ from 8.1→3 at 24 months, but PHN subgroup not reported separately	• 18 explants (loss of effect, infection)
Wang et al. (2023) [8]	Case report	N=1 (PHN=1)	• NRS: 8→2	• None
Kim et al. (2020) [16]	Case series	N=2 (PHN=2)	• Case 1: VAS 9→1 at 36 months. Case 2: 75–80% pain relief at 24 months	• None

Discussion

This scoping review synthesized current evidence regarding the effectiveness and safety of DRG stimulation for managing PHN. Overall, the evidence suggests that DRG stimulation may provide meaningful pain relief for PHN patients who have not responded adequately to conventional treatments. Many studies demonstrated significant reductions in pain scores and improvement in quality of life, often accompanied by decreased opioid and analgesic use.

From a clinical perspective, these findings are particularly relevant for pain specialists and multidisciplinary teams faced with challenging cases of refractory PHN. The potential for improved pain control and reduced reliance on systemic analgesics highlights DRG stimulation as an emerging option for select patients when standard treatments have failed. By mapping the spectrum of real-world outcomes and safety profiles reported to date, this review can help inform clinical decision-making regarding patient selection, expected benefits, and possible risks associated with DRG stimulation.

However, despite these promising findings, significant methodological limitations across the available literature restrict definitive conclusions. Most studies were retrospective, relied on small sample sizes, or consisted of case reports and series, reducing their external validity and generalizability. The literature was predominantly composed of retrospective analyses, case series, or isolated case reports. Common limitations included small sample sizes, heterogeneous patient populations, varied DRG stimulation parameters (e.g., percutaneous vs. surgical lead placements, tonic vs. burst vs. high-frequency stimulation), and a lack of long-term follow-up data. Although several studies provided quantitative assessments using validated tools such as the VAS and NRS, the lack of standardized outcome measures and consistent follow-up durations significantly complicates the interpretation and comparability of results. Additionally, many studies included mixed neuropathic pain etiologies rather than exclusively PHN, complicating direct comparisons and interpretation of outcomes specific to PHN patients.

Adverse events, although inconsistently reported, generally included lead migration, infection, skin erosion, and device failure or reduced efficacy requiring reprogramming or explantation. The variability in reporting these complications further complicated the reliability and comparability of safety outcomes.

The variability in DRG stimulation techniques, ranging from percutaneous lead placement to open surgical approaches and differences in stimulation programming (tonic, burst, or high-frequency stimulation), further complicates direct comparisons across studies. This heterogeneity makes it challenging to discern optimal DRG stimulation protocols, underscoring the need for standardized clinical guidelines to enhance future research consistency.

Another key limitation identified is the mixed-pain populations within studies, with only a subset explicitly addressing PHN. Including diverse neuropathic pain etiologies within the same studies dilutes the specificity of the findings regarding DRG stimulation effectiveness, specifically for PHN. Given the unique pathophysiological mechanisms underlying PHN, future research should aim for rigorous inclusion criteria that isolate this patient population to generate more targeted and reliable evidence.

Safety and complication profiles of DRG stimulation were inconsistently reported but frequently included adverse events such as lead migration, infection, and loss of therapeutic efficacy. Eldabe et al. (2022) notably reported that a significant proportion of patients required device revisions, emphasizing that, although DRG stimulation may offer significant benefits, potential complications must be carefully managed [24]. Consistent and detailed reporting of complications in future research is crucial for accurately balancing therapeutic efficacy against procedural risks.

Encouragingly, limited higher-quality evidence from RCTs, such as the studies by Parker et al. (2021) and Piedade et al. (2023), indicates that rigorous study designs are feasible and beneficial. These trials demonstrated significant reductions in pain and provided robust preliminary evidence of the potential superiority of DRG stimulation compared to placebo or sham conditions. Nevertheless, the limited number and small size of these trials highlight the urgent need for larger-scale RCTs [25,26].

Future research should prioritize addressing identified methodological gaps. Specifically, robust RCTs with larger, well-defined PHN cohorts, standardized DRG stimulation parameters, and longer follow-up periods are critically needed. Additionally, exploring comparative effectiveness studies, evaluating DRG stimulation against alternative neuromodulation techniques such as conventional SCS, could further elucidate optimal treatment paradigms for PHN patients.

In this context, the present review provides a practical summary for clinicians seeking to weigh the potential role of DRG stimulation within a multidisciplinary treatment strategy. While the data are not yet sufficient to support broad clinical recommendations, this synthesis may assist in identifying suitable candidates and facilitating informed discussions about expected outcomes and existing uncertainties.

Given the significant gaps and variability in the existing literature, this review serves as a foundational synthesis and pilot mapping of the available evidence on DRG stimulation for PHN. By systematically charting the landscape of published studies, this work lays the groundwork for future research, highlighting key areas of uncertainty and providing a baseline for subsequent, more definitive investigations. As one of the first comprehensive scoping reviews specifically focused on DRG stimulation in PHN, our study offers an important reference point for clinicians and researchers considering this therapy in refractory cases.

Future directions

The current evidence base for DRG stimulation in PHN is characterized by small studies, mixed patient populations, and a lack of standardized protocols, limiting both the strength and applicability of available data. As a result, clear research priorities have emerged. There is an urgent need for well-designed, prospective RCTs that focus exclusively on patients with PHN, employ uniform stimulation parameters, and utilize validated pain and quality-of-life measures with adequate long-term follow-up. Future investigations should aim to directly compare DRG stimulation with other established and emerging neuromodulatory or medical management strategies for PHN to clarify relative efficacy, optimal patient selection, and long-term outcomes.

Additionally, establishing consensus guidelines for the design, conduct, and reporting of neuromodulation studies in PHN would help enhance consistency, facilitate data synthesis, and accelerate clinical translation. Collaborative research efforts across multiple centers and the use of patient registries may further support the generation of robust, generalizable evidence. Ultimately, targeted and methodologically rigorous research is essential to advance our understanding of DRG stimulation for PHN and inform clinical decision-making.

## Conclusions

This scoping review serves as a foundational synthesis and pilot mapping of the current literature on DRG stimulation for PHN. Our findings highlight preliminary evidence that DRG stimulation may be a promising treatment modality for refractory PHN, with several studies reporting improvements in pain intensity and quality of life. However, the available evidence remains significantly limited by methodological variability, small and heterogeneous patient populations, inconsistent outcome reporting, and insufficient safety data, all of which preclude firm clinical recommendations at this time.

There is a pressing need for larger, high-quality RCTs focused specifically on PHN populations, utilizing standardized stimulation protocols, outcome measures, and robust safety reporting. Future research should also directly compare DRG stimulation to other neuromodulation and medical management strategies for PHN to better clarify its optimal role in clinical practice. Enhanced methodological rigor and targeted investigation will not only strengthen the existing evidence base but also lay the groundwork for the development of clear clinical guidelines. Ultimately, this pilot review underscores both the therapeutic potential and the current limitations of DRG stimulation for PHN, and provides an important reference point to guide future, more definitive studies in this evolving field.
